# Synchronous adenocarcinoma and carcinoid tumor of the terminal ileum in a Crohn's disease patient

**DOI:** 10.1186/1471-2407-5-157

**Published:** 2005-12-08

**Authors:** Ugo Cioffi, Matilde De Simone, Stefano Ferrero, Michele M Ciulla, Alessandro Lemos, Ettore Contessini Avesani

**Affiliations:** 1 Department of Surgery, University of Milan, Fondazione Ospedale Maggiore Policlinico, Mangiagalli e Regina Elena, IRCCS, Milano, Italy; 2 Department of Pathology, Azienda Ospedaliera San Paolo, Milano, Italy; 3 Istituto di Medicina Cardiovascolare, University of Milan, Fondazione Ospedale Maggiore Policlinico, Mangiagalli e Regina Elena, IRCCS, Milano, Italy; 4 Department of Radiology, Fondazione Ospedale Maggiore Policlinico, Mangiagalli e Regina Elena, IRCCS, Milano, Italy

## Abstract

**Background:**

Several malignancies have been described in association with inflammatory bowel diseases, the most common being adenocarcinoma. Carcinoid tumor and Crohn disease has also been previously reported, however the coexistence of both neoplasms is quite rare and the clinical diagnosis is very difficult. Here we report what we believe to be the fourth case of a mixed adenocarcinoid tumor coexisting with Crohn's disease.

**Case report:**

The patient presented with clinical and radiological features of intestinal obstruction. Laparotomy showed a stricturing lesion in the last 6 cm of the terminal ileum with proximal dilation. Only the histology of the resected surgical specimen proved the presence of a mixed adenocarcinoid tumor involving the terminal ileum.

**Conclusion:**

Carcinoid tumor should be suspected in elderly patients with Crohn's disease presenting with intestinal obstruction and laparotomy should be considered to exclude malignancy.

## Background

It has been recognized that patients with Crohn's disease (CD) are at increased risk of developing malignant lesions [1,2]. Adenocarcinoma is one of the most common malignant tumors of the small intestine complicating CD, even if it remains quite rare compared with the large bowel malignancies [3-5]. Carcinoid tumors have also been described in association with CD [6,7].

We report here a case of ileal Crohn's disease associated with both adenocarcinoma and carcinoid tumor.

## Case presentation

A 64-year old woman was admitted to the Gastroenterology Unit of another Hospital for abdominal pain, nausea, weight loss, and recurrent episodes of constipation. She had suffered from intermittent pain located in the right lower quadrant for 2 years, but denied chronic diarrhea. She was diagnosed as having Crohn's disease of the terminal ileum by ileocolonoscopy and histology. An upper gastrointestinal series excluded CD in the upper gastrointestinal tract.

**Figure 1:**
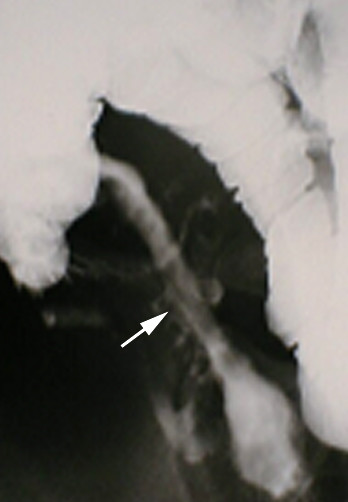
Small bowel double-contrast enteroclysis (detail) showing a stricture (arrow) of the terminal ileum with a thickening of the wall.

After inductive therapy with azathioprine and corticosteroids, the patient was maintained with mesalazine 500 mg × 4 daily. Prednisone 50 mg/day was added only during acute phases; in the six years following the discharge, 2 episodes of abdominal pain and dyarrhea requiring steroid therapy were recorded. Seven years later the patient was admitted to our Institution for abdominal pain, nausea, iron deficiency anemia, hypoalbuminemia, and intestinal obstruction. On physical examination no masses in the abdomen were noted. Small bowel double-contrast enteroclysis (Fig. 1) showed a stricture of the terminal ileum with a thickening of the wall without extravasation of the contrast material. CT scan performed by using intravenous and oral contrast material revealed thickening of the terminal ileum with stranding of the mesenteric fat. There was no clinical evidence of carcinoid tumor so biochemical tests for carcinoid syndrome were not carried out.

At operation, there was a stricturing lesion in the last 6 cm of the terminal ileum with proximal dilation. The wall of the affected ileal loop and caecum was thick and fibrous. Resection of the terminal ileum and caecum was performed. The intestinal continuity was obtained with an end-to-end ileocolic hand-sewn anastomosis.

**Figure 2:**
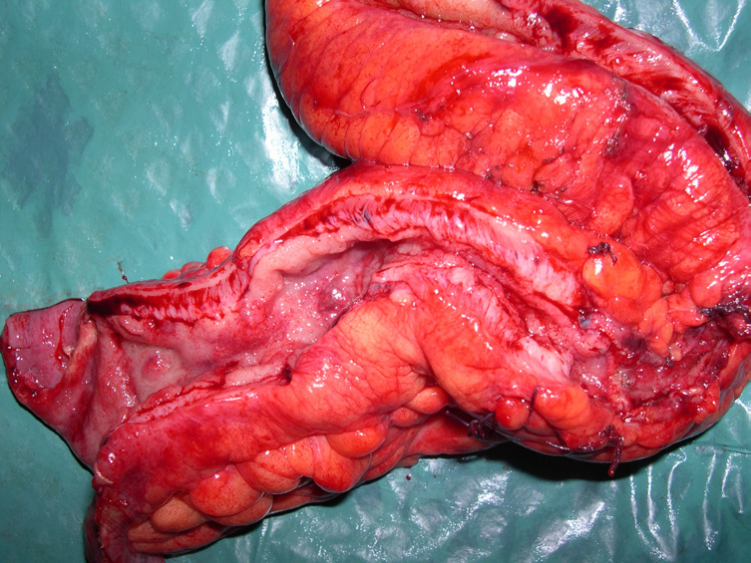
The ileal loop shows an important thickness of the wall with disappearance of the normal mucosal plicae.

**Figure 3:**
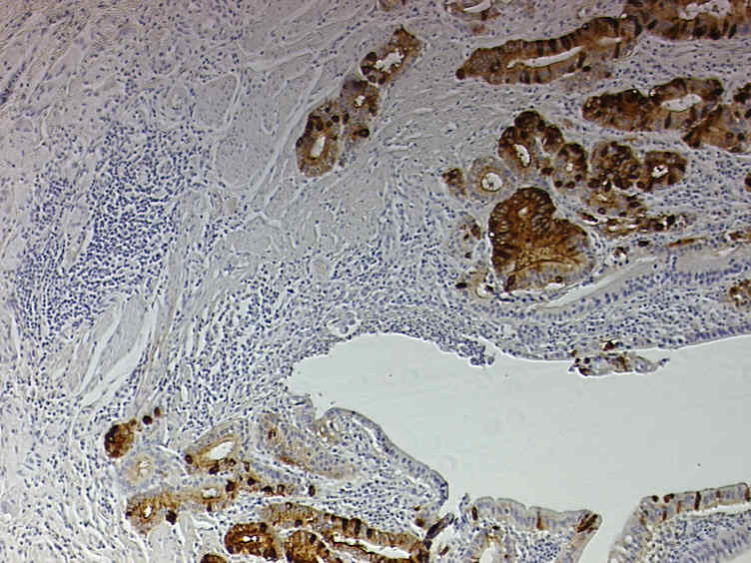
Strong immunoreactivity for Chromogranin A of carcinoid tumor infiltrating the muscular layer of the intestinal wall is clearly evident. The deep ulcer of CD is also present. (Magnification: ×25; Chromogen diaminobenzidine).

The surgical specimen included 28 cm of the terminal ileum and caecum. Grossly, all the length of surgical resection showed diffuse thickness of the intestinal wall. There was no evidence of malignancy (Fig. 2). Histologically, all resected surgical specimen was characterized by deep ulcers, marked proliferation of small lymph nodules involving all layers of the intestinal wall, sometimes with sarcoid-type granulomas and serosal inflammation. Moreover, two malignancies growing together were found in the ileal loop. The first showed glandular differentiation of large cells with round to oval nuclei sometimes with large nucleoli and abundant pale cytoplasm infiltrating the entire wall (pT2N0M0). The latter consisted of quite uniform medium size cells with round nuclei and inconspicuous nucleoli growing in a trabecular or acinar pattern (Fig. 3). Only the immunohistochemistry of this latter tumoral population showed strong and diffuse immunoreactivity to neuroendocrine markers such as Chromogranin A and Synaptophisin. All excised mesenteric lymph nodes were negative for malignant cells. The postoperative recovery was uneventful and the patient was discharged in general good condition. At 18-months follow-up the patient, checked by colonoscopy and ileoscopy was well, free from malignancy relapse or recurrence of CD, and with normal 24-hour urinary excretion of 5-hydroxyindoleacetic acid (5-HIAA).

## Discussion

Malignant tumors of the small intestine are very rare in the general population, so even the few cases reported in the Literature in association with CD are enough to consider CD patients at 4–20 fold increased risk for small bowel malignant neoplasm [3-5].

About 2% of patients affected by CD will develop cancer in the course of their disease, and, in contrast to the patients with ulcerative colitis (UC), those with CD are at risk for developing malignancy even in the first decade of their disease [5]. The coexistence of CD with adenocarcinoma is predominantly seen in men, in the patients with excluded loop, and most frequently in the distal ileum in an area of active disease [2,5]. Most patients presenting with adenocarcinoma complicating CD have a high-grade malignancy with lymph node involvement or distant metastases, because the similarity of the presenting symptoms and of the radiography of these pathologies creates diagnostic problems for the physician and make an early diagnosis impossible [3].

Until now, there is no epidemiological evidence that patients with CD are at increased risk for carcinoid tumor [8]. However, when associated with CD, carcinoid tumors tend to be malignant and have a worse prognosis [6]. The majority of carcinoid tumors are asymptomatic and discovered at histology. When symptomatic, they may cause pain due to the intestinal obstruction or perforation mimicking CD clinically and radiologically and creating difficulty in the differential diagnosis.

Only three cases of a coexistent CD and mixed adenocarcinoid tumor have been reported in the English Literature [8-10]. Ours is the fourth case in the Literature, and the third in which the CD and the mixed adenocarcinoid tumor were located in the terminal ileum. As in the three previous reported cases, the definitive diagnosis of mixed adenocarcinoid tumor associated with CD was possible only on histology of the resected surgical specimen. Despite the unfavourable prognosis, our patient is still alive and free from malignancy recurrence at 18-months follow-up.

Reviewing the Literature [1,6,7], we believe that an ileal carcinoid tumor should be suspected in elderly CD patients presenting with obstructive symptoms as in our case. Moreover, when technically feasible, an ileoscopy with biopsy may play a role in the differential diagnosis between CD and small bowel malignancy favoring a precise early diagnosis and a more appropriate treatment. However, laparotomy should immediately be considered in CD patients presenting a diagnostic dilemma.

## Competing interests

The author(s) declare that they have no competing interests.

## Authors' contributions

UC, MDS enrolled the patient for clinical and surgical aspects, drafting the article.

SF histological examinations.

MMC setting up images, critical revision of the article.

AL radiological imaging.

ECA performed surgical operations, critical revision of the article, final approval of the version

All Authors read and approved the final manuscript.

## Pre-publication history

The pre-publication history for this paper can be accessed here:
